# The effect of 2D finite element mesh properties on the dynamic behavior of saturated sands

**DOI:** 10.1038/s41598-026-59788-x

**Published:** 2026-06-26

**Authors:** Serdar Koltuk, Ozan Subasi

**Affiliations:** 1 Civil Engineering Department, Jade University of Applied Sciences, 26121 Oldenburg, Germany; 2 Faculty of Engineering, Civil Engineering Department, Çanakkale Onsekiz Mart University, Çanakkale, 17020 Turkey

**Keywords:** Dynamic Behavior, 2D Finite Element Analyses, Mesh Properties, Saturated Sands, PM4Sand, Engineering, Natural hazards, Solid Earth sciences

## Abstract

Numerical analyses are frequently used to determine the dynamic behavior of soils. In order to obtain correct results, it is extremely critical to establish a suitable finite element mesh. While excessively coarse meshes shorten the calculation time and cause details to be ignored, dense finite element meshes lead to prolonged analysis times and high costs. Therefore, the selection of the optimum finite element mesh density in numerical analyses is a critical issue for the balance between performance and efficiency. The present study studies the effect of 2D finite element mesh properties on the dynamic behavior of saturated sands. Three different sand soils with loose, medium and dense densities were modelled with the PM4Sand constitutive equation, and a total of 96 numerical analyses were performed for four different acceleration-time records. The findings of the analyses provide practical guidance for optimizing mesh selection in numerical analyses performed within the scope of geotechnical earthquake engineering.

## Introduction

Seismic waves generated during strong motion propagate through soil layers to the ground surface. Throughout their propagation, both the amplitude and frequency content of these waves change, while shear strains develop within the soil layers. During earthquakes, accurate estimation of the deformations in the near-surface soil layers, depending on the magnitude of the design motion in the bedrock, is of critical importance to minimize the effects on engineering structures.

In order to accurately predict the levels of deformation that may develop within soil layers during earthquakes, numerical analyses are conducted using soil profiles constructed by integrating laboratory test results with representative field conditions. The dynamic behavior of the stratified soil system is characterized through appropriate constitutive models, calibrated using data obtained from laboratory experiments. One, two or three-dimensional numerical simulations are then performed by applying acceleration time histories that are representative of the seismicity of the studied region^1–3^. In one-dimensional dynamic analyses, it is assumed that both seismic wave propagation and soil properties vary only in the vertical direction. The one-dimensional analyses yield conservative results if the affected soil layers extend horizontally to infinity and the layer boundaries can be assumed to be horizontal. Furthermore, the amplitude, frequency content, and duration, and thus the damaging effect, of two earthquakes can be very different, even if both earthquakes exhibit the same maximum seismic acceleration.

2D and 3D dynamical analyses, on the other hand, offer a more comprehensive modeling framework by accounting for the multi-directional components of strong ground motion and the spatial variability within soil layers. Although three-dimensional dynamic simulations provide the most realistic representation of in-situ soil behavior, they are not widely preferred in practice due to their high computational cost and the uncertainties associated with defining complex constitutive parameters. In this context, 2D analyses are commonly adopted as a good alternative, as they can effectively capture soil–structure interaction in two spatial directions while allowing for more feasible calibration and interpretation based on laboratory and field data^4–6^ Due to their ability to capture the time-dependent evolution of excess pore water pressure, two-dimensional simulations offer significant advantages in terms of both computational efficiency and practical applicability^7–9^.

These analyses typically follow four main steps: first, acceleration time histories are selected based on the objectives of the study and the seismic characteristics of the region; second, a suitable constitutive model is chosen to represent the soil behavior, and the associated parameters are calibrated using laboratory and/or field data. In the third stage, a finite element mesh appropriate for the soil profile is constructed, considering factors such as mesh density, element type, and boundary conditions. Finally, analysis scenarios are defined in accordance with the physical problem, and time-domain simulations are carried out. A critical aspect of this process is ensuring that the selected input motion propagates accurately through the soil domain via the defined finite element mesh, as this directly influences the reliability of the numerical results.

The mesh density of the defined geometric soil profile plays a critical role, particularly in accurately representing seismic wave propagation and precisely capturing the time-dependent evolution of excess pore water pressure. In numerical analyses, mesh density is commonly evaluated using the concept of “average element size,” and the accuracy of the analysis is largely governed by this parameter. The literature offers various recommendations for determining the appropriate average element size, along with criteria based on wave propagation characteristics—such as maintaining a specific ratio between the shortest wavelength and element size^10–12^. To minimize numerical dispersion and phase errors, it is essential that these criteria are satisfied. Accordingly, constructing a finite element mesh that is sufficiently refined to be compatible with the temporal resolution of the analysis, and selecting a constitutive model capable of realistically capturing the stress–strain behavior of the soil, are both fundamental to ensuring the reliability of two-dimensional saturated sand behavior simulations.

**Fig. 1:**
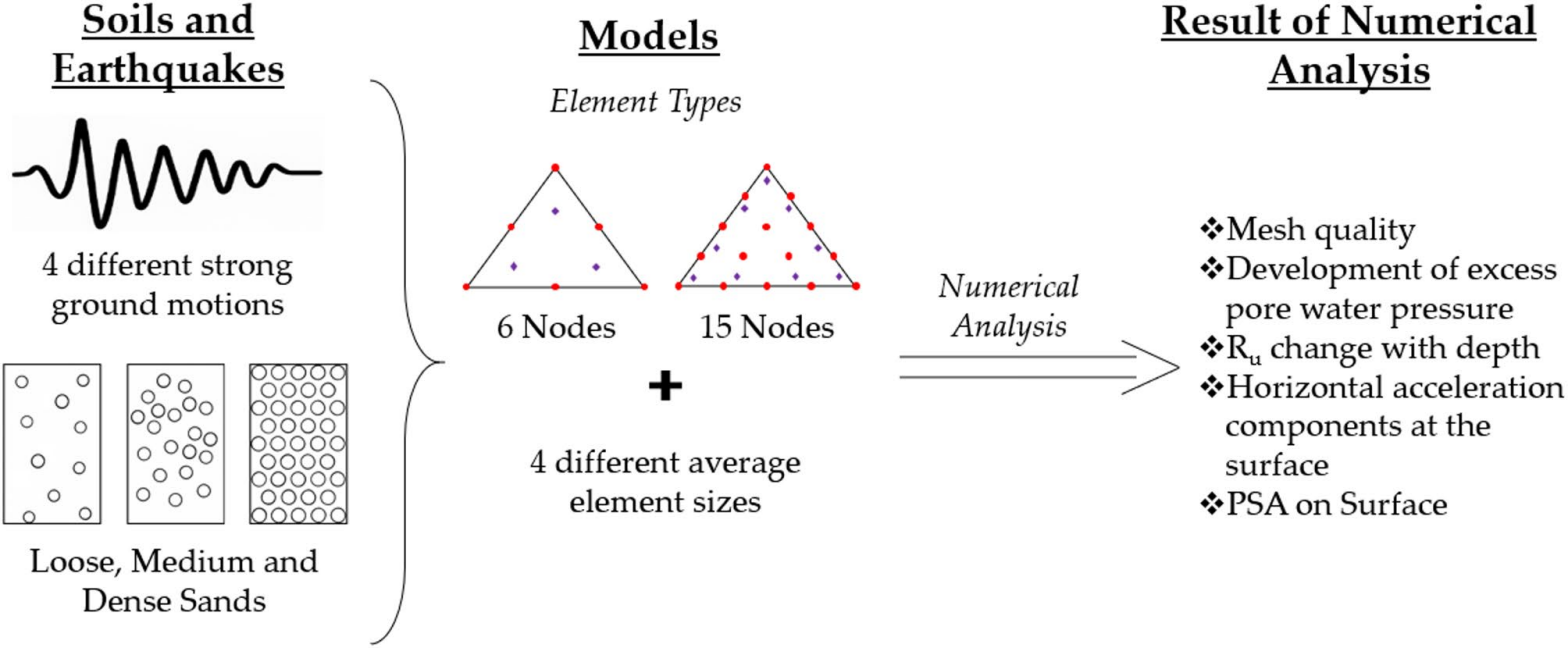
The graphical abstract of this study.

In this study, a series of two-dimensional numerical analyses were performed by using the FE method to systematically investigate the influence of finite element mesh properties on the dynamic behaviour of saturated sands. By comparing the simulation results, the effect of finite element mesh density on the accuracy and reliability of saturated sand behavior was evaluated in detail. Figure 1 provides a visual summary of the scope and methodology of the present study.

## 2D finite element analyses

The soil profiles were defined using the properties of loose, medium-dense, and dense sands, and their dynamic behavior was modeled with the PM4Sand constitutive model in the FE Software Plaxis 2D^13^. To assess the effect of mesh refinement on the simulation outcomes, four different mesh configurations were created using both 6-nodes and 15-nodes triangular elements. Dynamic analyses were conducted using four acceleration time histories selected to represent different frequency contents. In all simulations, the geometry, boundary conditions, and loading application were kept constant to isolate and evaluate the effect of mesh properties on the key response parameters, such as excess pore pressure, surface horizontal acceleration and pseudo-spectral acceleration.

### Constitutive model

The PM4Sand constitutive model developed by Boulanger and Ziotopoulou is capable of capturing the nonlinear and cyclic response characteristics of granular materials. It is a stress ratio-controlled, critical state-compatible model based on bounding surface plasticity theory, and is widely applied in geotechnical earthquake engineering^14^. The model successfully represents essential features of saturated sands, including cyclic mobility, dilatancy, and the generation of excess pore water pressure, making it particularly suitable for liquefaction analysis in clean sandy soils^15–17^. The PM4Sand model parameters adopted in this study for different relative densities of clean Ottawa sands are presented in Table 1^14,18^.

However, due to the limitations of PM4Sand in accurately representing the initial stress conditions and small-strain stiffness behavior, the Hardening Soil model with small-strain stiffness (HSSM) was employed to simulate the pre-seismic response of the soil domain^19,20^. The HSSM model enhances the classical Hardening Soil formulation by incorporating strain-dependent stiffness degradation, which is crucial for establishing realistic initial stress distributions prior to dynamic excitation. The model parameters were calibrated based on relative density, and the input values are summarized in Table 2^21^.

**Table 1:** PM4Sand constitutive parameters for different relative densities^14,18^.

Symbol	Description	Unit	D_*r*_=35%	D_*r*_=55%	D_*r*_=75%
D_r_	Relative density	–	0.35	0.55	0.75
γ_dry_	Dry unit weight	kN/m³	15.34	15.9	16.52
γ_sat_	Saturated unit weight	kN/m³	19.36	19.71	20.1
e	Void ratio	–	0.695	0.635	0.575
G_0_	Small-strain shear modulus	MPa	476	677	890
h_p₀_	Hardening exponent parameter	–	0.53	0.4	0.63
e_max_	Maximum void ratio	–	0.80	0.80	0.80
e_min_	Minimum void ratio	–	0.50	0.50	0.50
P_a_	Reference pressure (atmospheric)	MPa	0.101	0.101	0.101
n^b^	Exponent for bulk modulus scaling	–	0.50	0.50	0.50
n^d^	Exponent for unloading/recompression stiffness	–	0.10	0.10	0.10
φ_cv_	Critical-state friction angle	°	33.00	33.00	33.00
ν	Poisson’s ratio	–	0.30	0.30	0.30
Q	Critical state parameters	–	10	10	10
R		–	1.5	1.5	1.5
PostShake	Specifies the drainage condition. It takes a value of 0 under undrained conditions and 1 under drained conditions.				

**Table 2:** Hardening small-strain model calibration and parameters for different relative densities^21^.

Symbol	Description	Method	Unit	D_*r*_=35%	D_*r*_=55%	D_*r*_=75%
γ_dry_	Dry unit weight	Ottawa soil properties [14], [18]	kN/m³	15.34	15.9	16.52
γ_sat_	Saturated unit weight		kN/m³	19.36	19.71	20.1
e	Void ratio		–	0.695	0.635	0.575
E_50ref_	Secant elasticity modulus	60,000×D_r_/100	MPa	21	33	45
E_oedref_	Tangent elasticity modulus	60,000×D_r_/100	MPa	21	33	45
E_urref_	Unloading–reloading elasticity modulus	180,000×D_r_/100	MPa	63	99	135
m	Stress-dependency exponent	0.7–D_r_/320	–	0.59	0.53	0.47
c’	Effective cohesion	Ottawa soil properties [14]	MPa	0	0	0
φ′	Effective friction angle		°	33	33	33
γ_0.7_	Shear strain at 0.7 G₀	(2–D_r_/100)×10⁻⁴	–	0.00017	0.00015	0.00013
G_0ref_	Reference small-strain shear modulus	60,000 + 68000D_r_/100	MPa	83.8	97.4	111
ν	Poisson’s ratio	Default value	–	0.3	0.3	0.3
P_ref_	Reference pressure (atmospheric)	Standard value	MPa	0.1	0.1	0.1
R_f_	Reduction factor	1–D_r_/800	–	0.956	0.931	0.906

### Strong ground motions

**Fig. 2:**
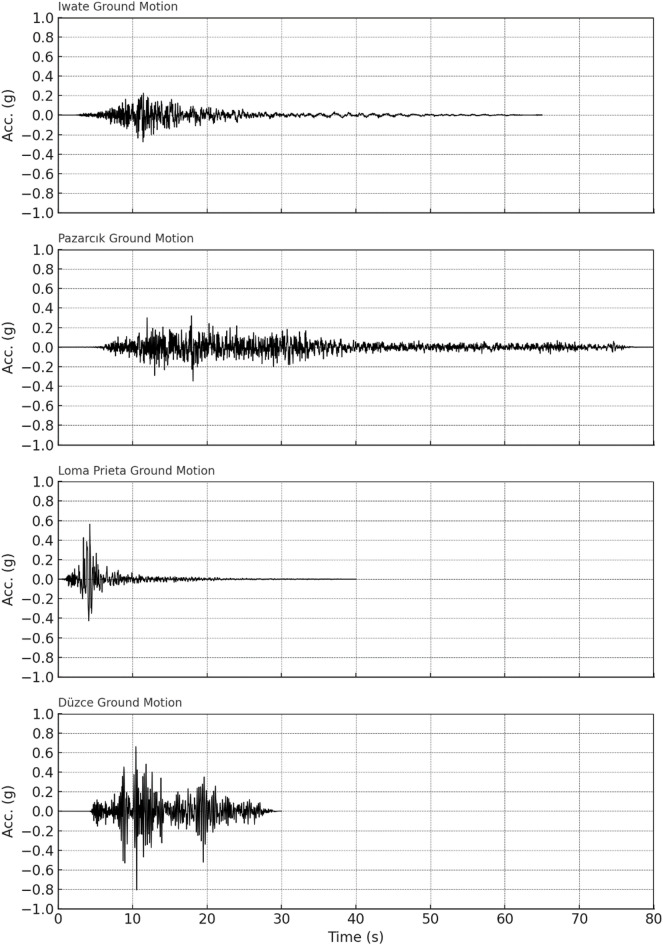
Acceleration-time histories of selected earthquakes.

The selection of strong ground motion records is a crucial step in numerical analyses aimed at evaluating soil behavior under dynamic loading conditions. The chosen acceleration time histories must be representative of the seismicity and potential source characteristics of the study area. In this study, four different acceleration records were selected from outcropping bedrock motions with varying frequency content and source characteristics to investigate the influence of finite element mesh density on the dynamic response of saturated sandy soils. The earthquake records used in the numerical simulations are illustrated in Fig. 2, while the key properties of the selected strong ground motions are summarized in Table 3. These records were obtained from the Pacific Earthquake Engineering Research Center (PEER) ground motion database to ensure consistency with well-documented and high-quality seismic input data^21^.

To ensure consistency across different mesh densities and soil profiles, the same set of ground motion records was used in all dynamic analyses. This approach eliminates variability arising from the input motions, allowing the effects of mesh refinement on the analysis results to be isolated and objectively evaluated. Each selected record was assessed in terms of its frequency content using Fourier and spectral analyses, and care was taken to include motions with distinct frequency characteristics. Consequently, the adopted ground motion set offers both strong representativeness and reproducibility, enabling a reliable investigation of the dynamic behavior of sandy soils.

**Table 3:** Characteristics of selected acceleration-time histories^21^.

No	Eartquakes	Stations	Fault Rupture Mechanism	V_s30_ (m/s)	M_w_	PGA (g)	Duration (s)	f_eq_ (Hz)
1	Iwate	IWT010	Reverse	826	6.90	0.27	65.00	0.59
2	Pazarcık	4611	Strike Slip	731	7.80	0.35	80.00	1.55
3	Loma Prieta	Gilroy Array#1	Reverse Oblique	1428	6.93	0.56	40.00	2.69
4	Düzce	IRIGM 496	Strike Slip	760	7.14	0.81	30.00	4.97

In the numerical analyses, the seismic loading was applied along the base of the model as a prescribed displacement of 0.5 m in the x-direction due to the compliant base condition, which means that reflected waves are damped, used in the numerical model. The vertical component of the motion in the y-direction was constrained using fixed boundary conditions, effectively eliminating displacement in that direction. This approach allows for a more realistic representation of both the strong ground motion and the dynamic soil response.

Equally important as the selection of the input motion is the time increment with which the acceleration record is applied to the soil profile. To prevent a seismic wave from traveling through multiple finite elements within a single time step, the critical time step (Δt) is determined using Eq. 1^13^.

1 $$\Delta t \leq \frac{l_{\min}}{V_s}$$

In this expression, l_min_ denotes the shortest distance between two adjacent nodes in the mesh, and V_s_ is the shear wave velocity of the soil layer. For the soil profiles developed in this study, the critical time step was set to 0.005 s. The strong ground motion records were resampled accordingly to ensure compatibility with this time resolution. This ensured numerical stability and preserved the accuracy of wave propagation throughout the dynamic simulations.

### Geometry of the numerical model and mesh properties

The geometry of the soil model and the properties of the finite element mesh play a critical role in influencing the outcomes of numerical analyses. In dynamic analyses, where the accurate representation of wave propagation and pore pressure buildup is essential, insufficient mesh density may lead to computational inaccuracies and underestimation of key response parameters. Thus, selecting an appropriate mesh density, in conjunction with a representative soil geometry, is essential for producing reliable and physically meaningful results.

In this study, the vertical extent of the soil profile was defined as 20 m, considering the typical thickness of liquefiable sand layers reported in the literature^22^. The horizontal extent was set to approximately five times the vertical length to minimize the influence of boundary conditions on the analysis results. The dimensions of the constructed soil model are consistent with geometric criteria proposed in previous studies and ensure a more realistic simulation of seismic wave propagation^23,24^.

**Fig. 3:**
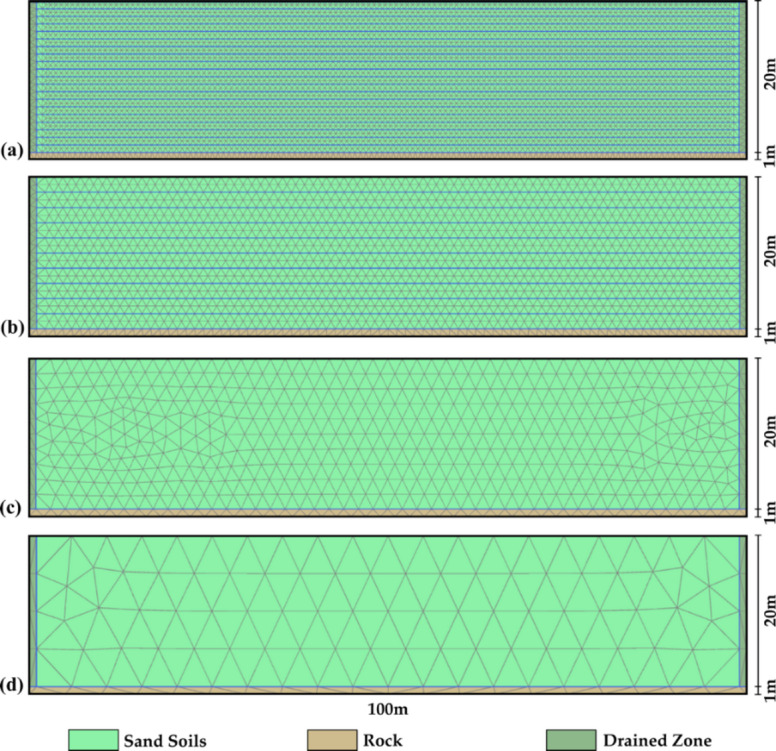
Finite element mesh densities **a**) Model 1, **b**) Model 2, **c**) Model 3, **d**) Model 4.

In the constructed soil profile, the upper layer consists of sand defined using properties corresponding to three different relative densities (D_r_ = 35%, 55%, and 75%). This soil geometry enables a systematic evaluation of how variations in mesh density influence the dynamic behavior of sands with different levels of compactness. Below the sand layer, an engineering bedrock layer with a thickness of one meter is placed, as shown in Fig. 3. This layer corresponds to ground type B as classified in the NEHRP and ensures realistic transmission of seismic waves into the soil body^25^. The rock mass was modeled as a linear elastic material according to the B soil type. In order to maintain compatibility with the boundary conditions used in the numerical model, drainage layers with similar properties to the sand were added along both lateral sides of the domain, each having a thickness of one meter.

In dynamic finite element analyses of geotechnical systems, the accuracy of wave propagation modeling is closely tied to the density of the mesh. An inappropriate element size may result in artificial dispersion, numerical damping, and phase distortion, which can compromise the reliability of predicted responses such as excess pore pressure generation and seismic settlements. For this reason, several researchers have proposed empirical and theoretical criteria to guide the selection of average element size (AES) in wave propagation problems.

Among the most frequently cited criteria in this context is the one proposed by Kuhlemeyer and Lysmer^10^. These researchers emphasized that, in order to minimize numerical errors in dynamic simulations, AES should be less than one-eighth of the wavelength associated with the maximum frequency of the input motion. This recommendation has since become a widely accepted guideline in both academic research and practical engineering applications, and is commonly expressed by Eq. (2):

2 $$AES \leq \frac{V_{s,\mathrm{layer}}}{8f_{\max}}$$

Here, V_s, layer_ is the shear wave velocity of the soil layer and f_max_ is the maximum significant frequency of the input acceleration record.

Later studies proposed revisions to the original criterion. For example, Greef showed that using higher order elements with eight or more nodes allows for acceptable accuracy even when larger element sizes are adopted^11^. This more flexible limit is showed in Eq. (3):

3 $$AES \leq \frac{V_{s,\mathrm{layer}}}{2f_{\max}}$$

In more recent work, Toloza introduced an alternative criterion that builds upon the earlier formulation by previous studies^12^. This approach is considered more suitable for practical engineering purposes as it offers a well-balanced compromise between computational efficiency and numerical accuracy. Equation (4) has been shown to be particularly effective in large-scale dynamic analyses of fully saturated sandy soils, where both precision and performance are critical:

4 $$AES \leq \frac{V_{s,\mathrm{layer}}}{5f_{\max}}$$

In this study, four distinct finite element mesh configurations were developed using 6-nodes and 15 nodes triangular elements, based on the mesh density criteria summarized earlier from the literature. These mesh schemes were applied to sandy soil profiles with relative densities of 35%, 55%, and 75%, and were evaluated under the same set of strong ground motion records to ensure consistency. Model 1 was designed with the smallest average element size to serve as a high-resolution benchmark. Model 2 was generated following the wave propagation criterion proposed by Kuhlemeyer and Lysmer. Model 3 was established based on the engineering-optimized formulation introduced by Toloza, which aims to balance computational cost and accuracy. Lastly, Model 4 was constructed according to the recommendation of Greef, which allows for larger element sizes when using higher-order elements. In order to obtain a mesh density that complies with the average element size equations proposed in the literature, the soil profile was divided into sublayers. Model 1 was constructed using sublayers with a thickness of 1 m, whereas Model 2 included sublayers with a thickness of 2 m. For Model 3 and Model 4, the soil profile was modeled as a single continuous layer without any sublayer. Representative illustrations of the mesh configurations are provided in Fig. 3, and detailed information on mesh characteristics is summarized in Table 4.

**Table 4:** Finite element mesh configurations.

Model No	6 Nodes			15 Nodes		
	AES (m)	#Elements	# Nodes	AES (m)	#Elements	# Nodes
Model 1	0.70	10,513	21,715	0.70	10,513	85,478
Model 2	1.21	3416	7266	1.21	3416	28,192
Model 3	2.13	1107	2482	2.13	1107	9386
Model 4	4.92	218	552	4.92	218	1972

### Boundary, drainage and damping conditions

The bottom boundary of the model was defined using a compliant base condition. Free-field boundary conditions were applied only along the lateral boundaries to represent the far-field soil response and to minimize artificial wave reflections from the vertical boundaries. The ground surface was modeled as a free surface, allowing the surface acceleration response to develop naturally during seismic loading. This combination of a compliant base, lateral free-field boundaries, and a free ground surface is commonly used in dynamic geotechnical analyses to improve the representation of seismic wave propagation and reduce boundary-induced numerical artifacts^26,27^. Alongside the mechanical boundaries, appropriate drainage conditions were incorporated to reflect the fully saturated nature of sandy soils during seismic excitation. The groundwater table was positioned at the ground surface, and the soil domain was assumed to remain fully saturated throughout the depth of the liquefiable layer. To achieve a realistic hydraulic response and prevent numerical artifacts related to pore pressure accumulation at the boundaries, lateral drainage zones with a thickness of one meter were introduced at both vertical edges of the model, as shown in Fig. 3. These drainage layers were assigned hydraulic properties consistent with those of the sandy soil. The combination of mechanical and hydraulic boundary conditions provided a robust framework for accurately capturing the dynamic response of the saturated soil system during earthquake loading^28^. Energy dissipation during strong ground shaking results from several mechanisms such as soil viscosity, internal friction, and irreversible shear deformations. Although plasticity-based constitutive models capture part of this behavior through hysteretic damping, they often fall short of representing the total damping observed in real soils. To overcome this limitation, Rayleigh damping parameters were introduced in PLAXIS 2D as an additional damping mechanism^29^. The Rayleigh damping matrix is formulated as the sum of the mass and stiffness matrices, as given in Eq. 5:

5 $$[C] = \alpha [M] + \beta [K]$$

In this expression, the coefficients alpha and beta are determined based on two target frequencies. The first frequency (f_1_) represents the fundamental frequency of the soil profile and is calculated using the average shear wave velocity and the thickness of the soil layer:

6 $$\:{f}_{1}={V}_{s,average}/4H$$

The second frequency (f_2_) is obtained by dividing the effective frequency content of the input ground motion by the first frequency and rounding the result to the nearest odd integer:

7 $$\:{f}_{2}={f}_{eq}/{f}_{1}$$

A series of dynamic analyses were performed using different target damping ratios to evaluate their influence on the excess pore water pressure response and liquefaction-related behavior. Based on the overall response trends and numerical stability of the analyses, a target Rayleigh damping ratio of 2% was selected for all cases, as it provided a realistic response while avoiding excessive artificial damping. The same 2% target damping ratio was applied to all mesh configurations in order to isolate the influence of average element size and element order. In other words, the prescribed damping was not varied with mesh density, so that the differences observed among Models 1 to 4 could be attributed primarily to the discretization characteristics rather than to different damping assumptions. This approach is consistent with the objective of the study, which is to evaluate the effect of mesh properties under otherwise identical numerical conditions.

### Numerical steps

In order to simulate the mechanical behavior of saturated sands with high accuracy, a sequence of analysis steps was defined. The first step involved initializing the in-situ stress conditions using the K_₀_ procedure. In the second step, the Hardening Soil model with small-strain stiffness was assigned to capture a realistic initial stress distribution. This approach was necessary because the PM4Sand model, although highly effective under dynamic conditions, lacks robustness for static loading. To ensure equilibrium prior to dynamic loading, this intermediate stage was introduced. In the final step, dynamic analyses were conducted using the PM4Sand constitutive model.

The development of excess pore pressure was captured using the ‘Undrained A’ condition. In PLAXIS 2D, the Undrained A option represents an effective-stress-based undrained formulation in which the soil stiffness and strength parameters are defined in terms of effective stresses, while excess pore water pressures are generated internally during undrained loading. This option was adopted because the sand layer was modeled as fully saturated and subjected to rapid seismic loading, for which drainage during shaking is limited. Therefore, the Undrained A formulation is consistent with the PM4Sand constitutive model and is suitable for evaluating excess pore pressure accumulation and liquefaction-related response under earthquake loading^28^.

## Results and discussion

This section presents and interprets the results of two-dimensional dynamic finite element analyses conducted on saturated sand profiles with varying relative densities. The primary objective is to examine how mesh density and element type influence the accuracy and physical realism of the numerical predictions related to dynamic soil behavior. By evaluating multiple mesh configurations across different relative densities, the study assesses the impact of numerical discretization on key output parameters. The discussion is organized into five focal areas: mesh quality, the development of excess pore water pressure, the variation of excess pore water pressure ratio with depth, horizontal acceleration at the ground surface, and surface response spectra. These aspects are essential for judging the reliability of numerical simulations in geotechnical earthquake engineering. Particular attention is given to understanding the trade-off between computational efficiency and result accuracy, as finer meshes typically provide more detailed insights into wave propagation and soil response but also demand greater computational resources. The results are interpreted in the context of practical engineering applications, highlighting the importance of selecting an appropriate mesh density and element formulation in order to achieve physically meaningful, reliable, and computationally manageable outcomes.

### Quality of mesh

The accuracy of results obtained from dynamic finite element analyses largely depends on the realistic propagation of the prescribed acceleration time history within the soil profile. Therefore, defining a mesh with appropriate density plays a critical role in ensuring accurate modeling of wave transmission and achieving reliable numerical outputs. In this context, a careful evaluation of mesh quality prior to interpreting the results is essential. Selecting the most suitable mesh refinement for the specific application not only enhances numerical stability but also ensures the physical relevance and trustworthiness of the simulated soil response.

**Fig. 4:**
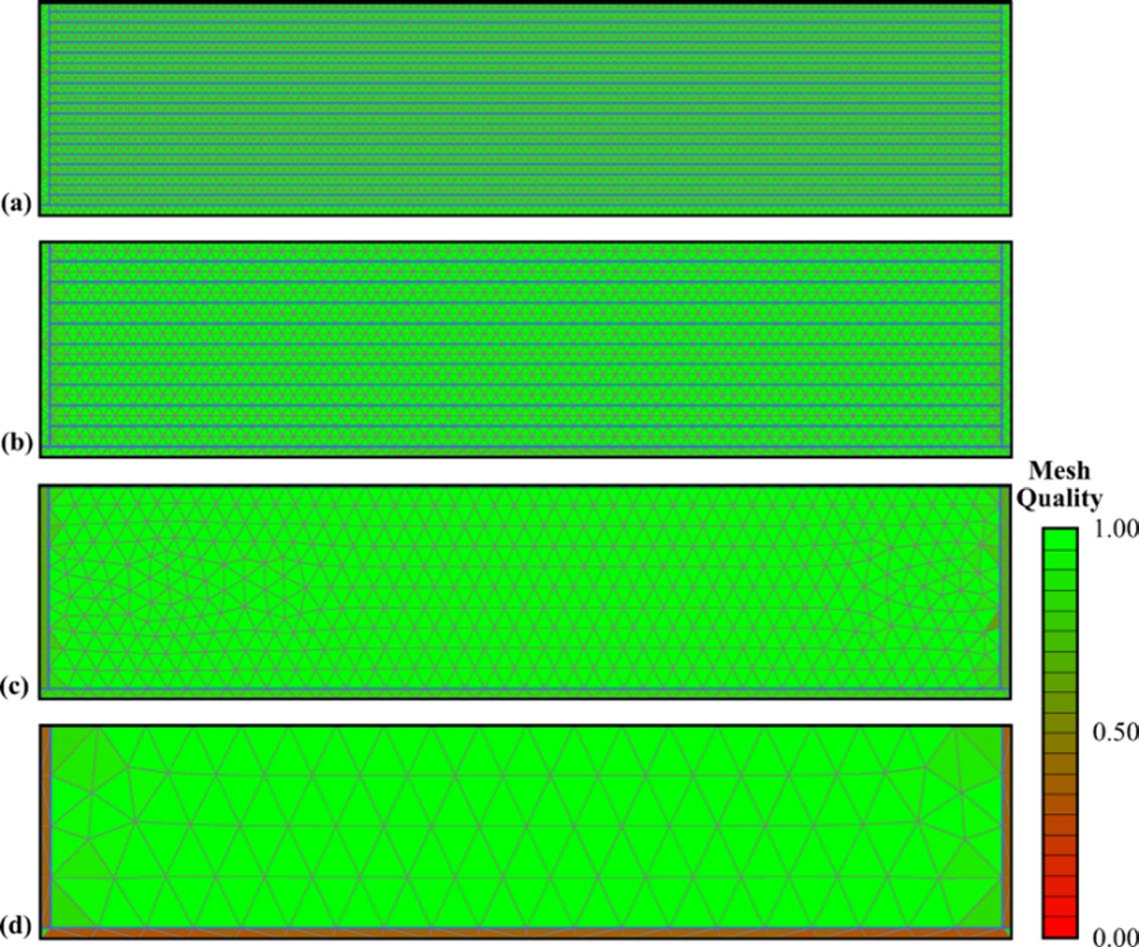
Quality of finite element mesh densities: **a**) Model 1, **b**) Model 2, **c**) Model 3, **d**) Model 4.

Figure 4 presents the mesh quality outcomes obtained for the four different finite element mesh densities defined in the scope of this study. Across all models, from Model 1 to Model 4, comparable levels of mesh quality were achieved for both 6 and 15 nodes triangular elements. Furthermore, the results indicate that different relative density values did not cause significant variation in overall mesh quality.

The mesh quality visualizations illustrated in the figure provide a quantitative means to assess the geometric fidelity and numerical suitability of the different finite element configurations. The associated color scale ranges from 0 to 1, where values approaching 1 are represented by green tones, indicating well-shaped and high-quality elements. Conversely, red tones highlight elements with distorted geometries and reduced shape quality. Models 1 and 2 demonstrate consistently high mesh quality throughout the domain, supporting their potential to yield reliable numerical results. In contrast, Models 3 and particularly Model 4 show significant deterioration in mesh quality within the boundary regions defined as drainage zones. Moreover, in Model 4, poor-quality elements are also observed within the bedrock zone, suggesting that excessively coarse meshes may compromise geometric consistency and hinder the accurate simulation of seismic wave propagation. This section has focused on evaluating the mesh quality for the four mesh configurations used. The subsequent sections provide a detailed examination of how these configurations influence key analysis results.

### Development of excess pore water pressure

**Fig. 5:**
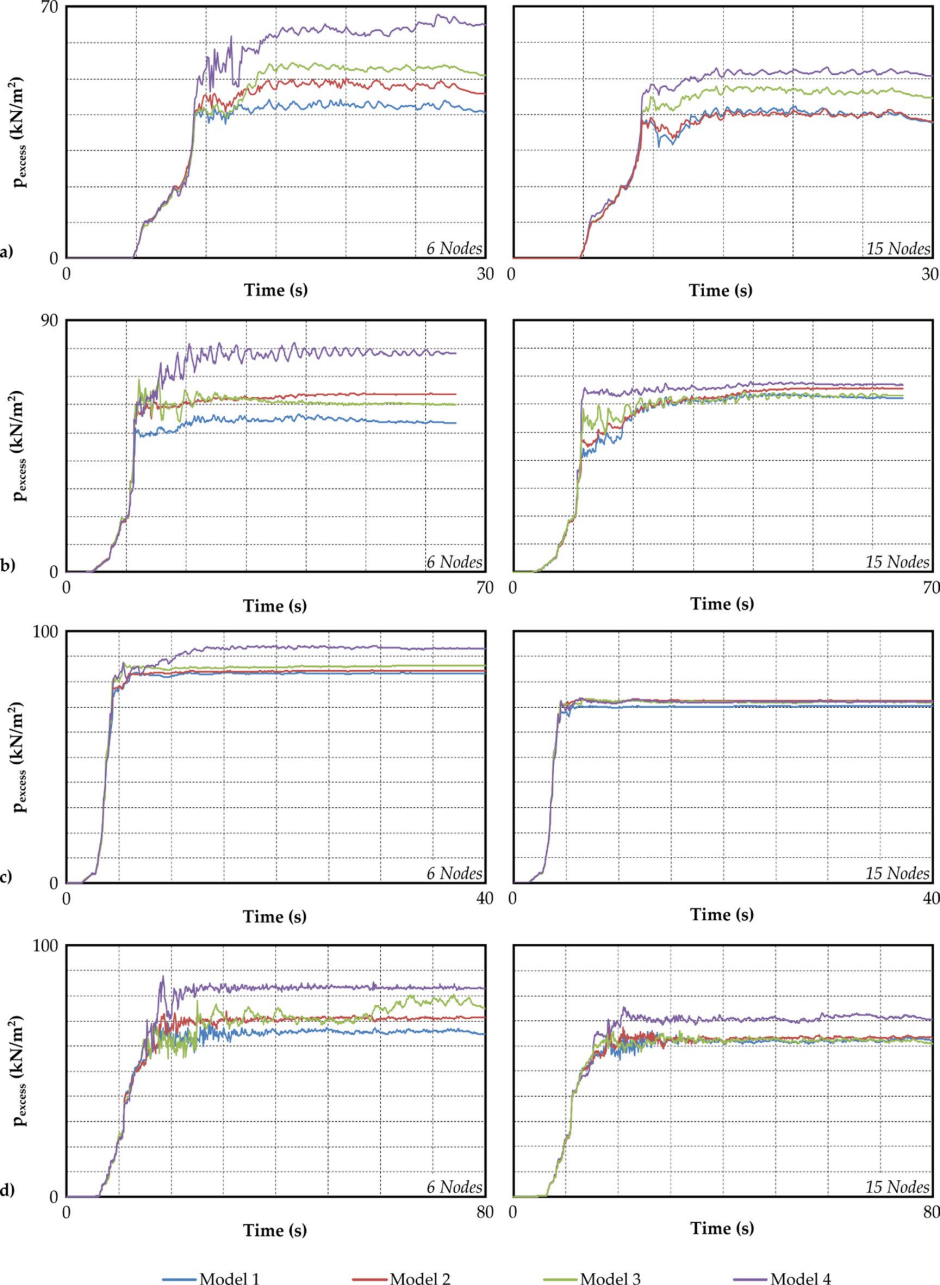
Effect of element type and mesh density on the development of excess pore water pressure in sand with 35% relative density under earthquake loadings: **a**) Düzce, **b**) Iwate, **c**) Loma Prieta, **d**) Pazarcık.

**Fig. 6:**
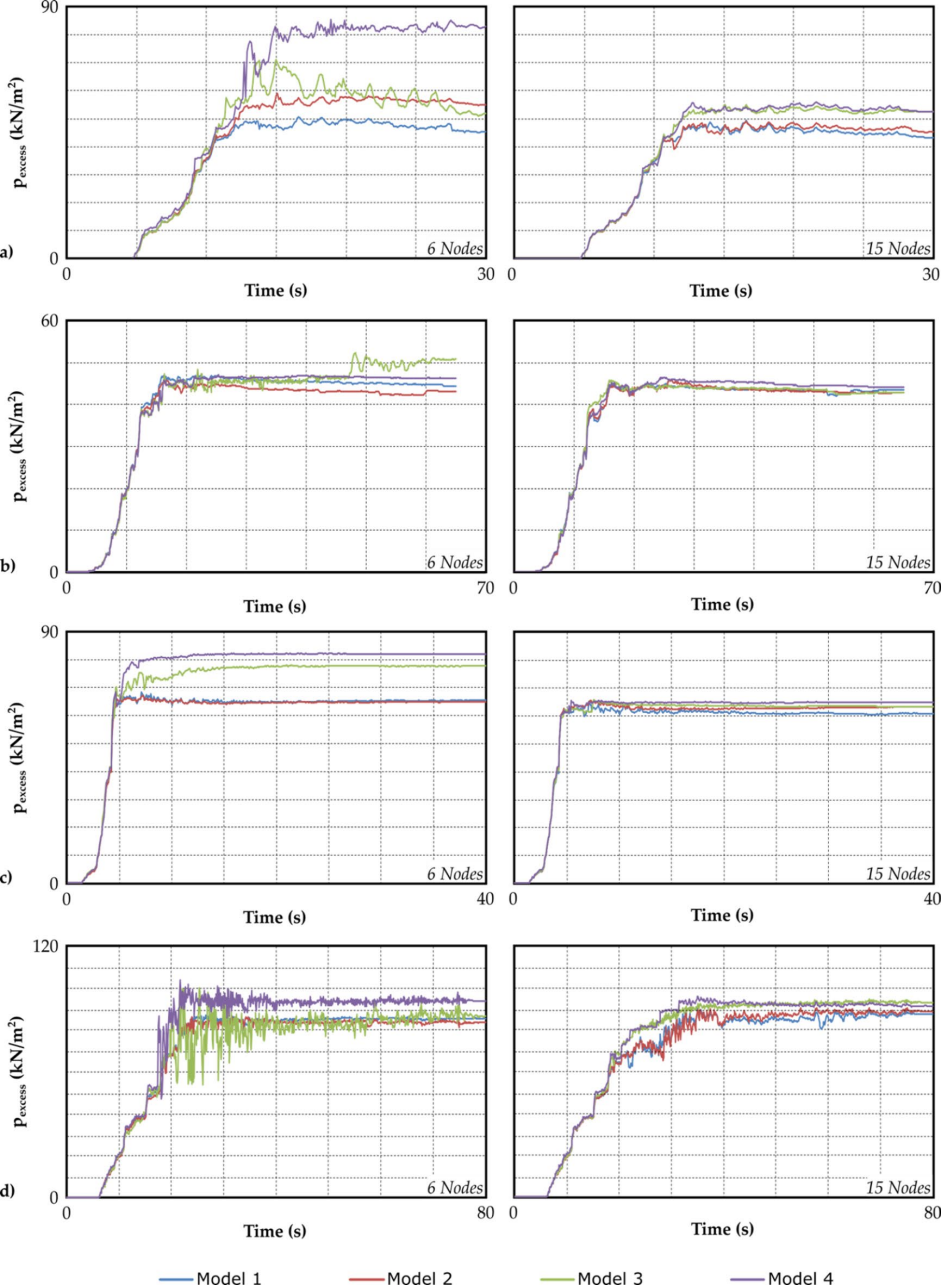
Effect of element type and mesh density on the generation of development pore water pressure in sand with 55% relative density under earthquake loadings: **a**) Düzce, **b**) Iwate, **c**) Loma Prieta, **d**) Pazarcık.

**Fig. 7:**
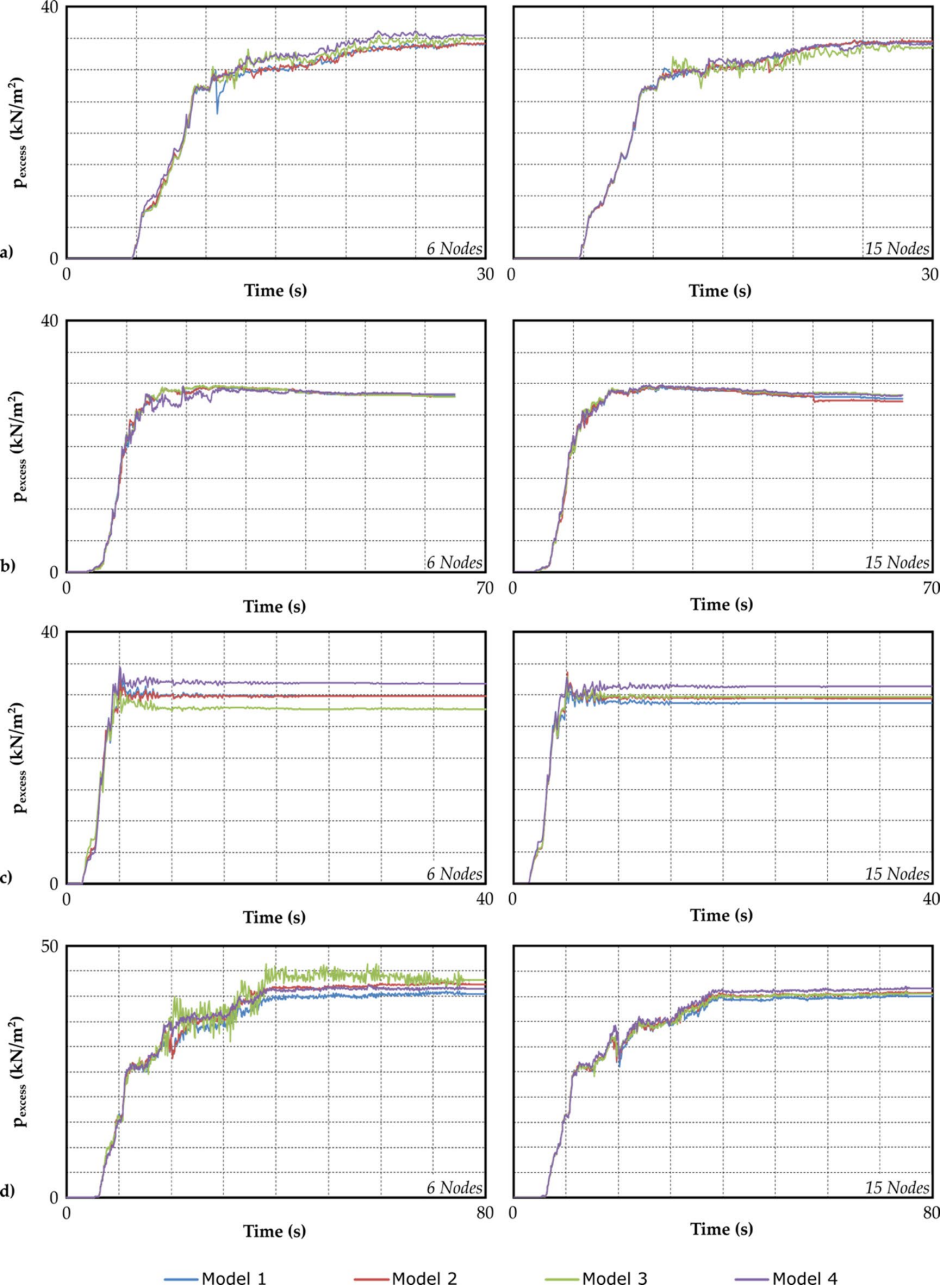
Effect of element type and mesh density on the development of excess pore water pressure in sand with 75% relative density under earthquake loadings: **a**) Düzce, **b**) Iwate, **c**) Loma Prieta, **d**) Pazarcık.

This section provides a detailed examination of the development of excess pore water pressure (p_excess_) obtained from two-dimensional dynamic analyses. For each earthquake record, measurements were taken at the node situated at mid-width of the model and ten meters below the ground surface to capture the development of the excess pore pressure response during strong motion. The excess pore water pressure responses obtained from all mesh density models, covering different seismic records and both 6 and 15 nodes elements at relative densities of 35, 55 and 75%, are presented in Figs. 5, 6 and 7, respectively.

The results indicate that development excess pore water pressure varies with the seismic source characteristics, the soil’s relative density, and the finite element mesh density. In general, the finest mesh (Model 1) produces the lowest peak pressures, whereas the coarsest discretization (Model 4) yields the highest peak values. When using 6 nodes triangular elements, increasing the relative density leads to a reduction in the difference between the responses of Model 4 and Model 1, although noticeable discrepancies persist at lower density levels. By contrast, when 15 nodes triangular elements are employed, even a coarse mesh configuration yields pressure time histories that closely match those of Model 1 across all density cases. Given that Model 1 consistently delivers the most reliable predictions, it is recommended to adopt a coarser mesh design in combination with 15 nodes elements rather than rely solely on the finest discretization. This strategy can significantly reduce computational time and memory requirements while preserving predictive accuracy.

### R_u_ change with depth

In numerical simulations of seismic soil response, the onset and progression of liquefaction are most reliably quantified through the excess pore water pressure ratio (R_u_), which reflects the loss of effective stress under cyclic loading. Accurately capturing how R_u_ varies with depth is essential for identifying potential liquefiable zones. In this study, we employ two-dimensional finite element models with systematically varied mesh densities and element type (6 and 15 nodes) to evaluate how spatial resolution and interpolation order affect R_u_ predictions across representative sand deposits. Dynamic loading is applied using four well-characterized earthquake records, and results are compared for three relative densities (35, 55 and 75%).

**Fig. 8:**
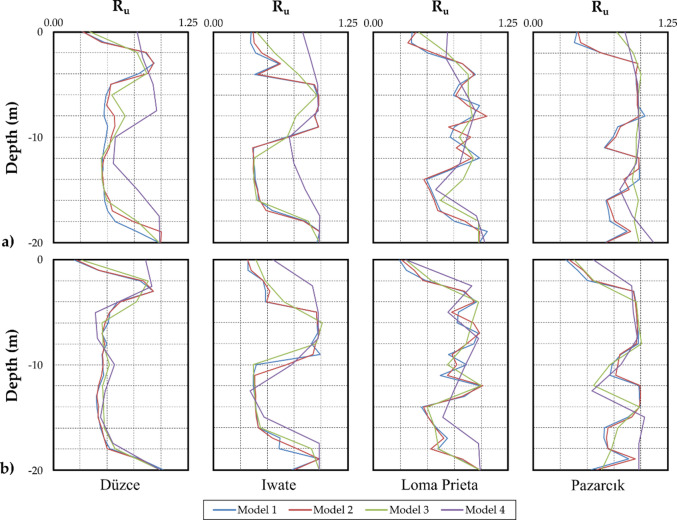
Variation of R_u_ with depth for a relative density of 35%: **a**) 6 nodes, **b**) 15 nodes.

**Fig. 9:**
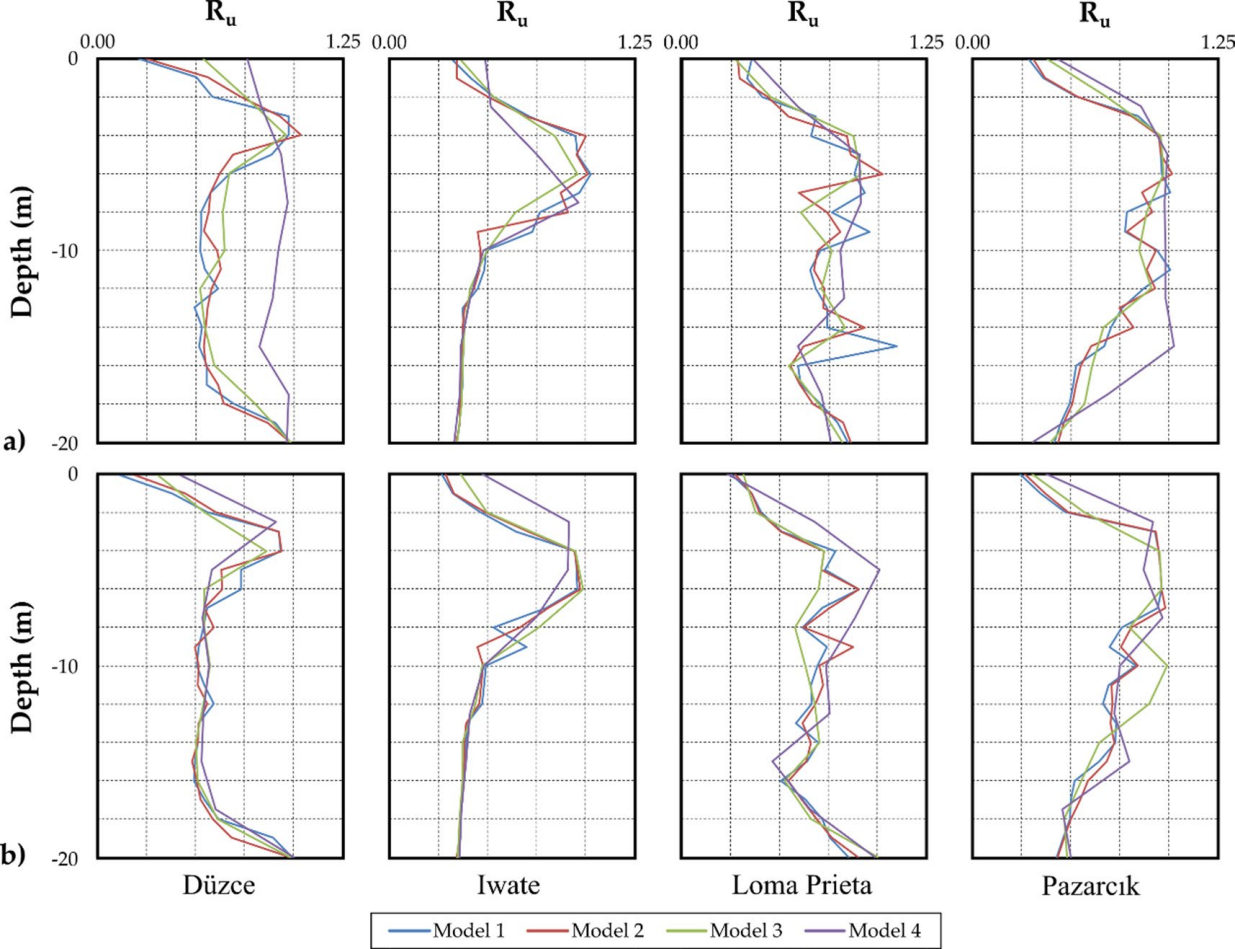
Variation of R_u_ with depth for a relative density of 55%: **a**) 6 nodes, **b**) 15 nodes.

**Fig. 10:**
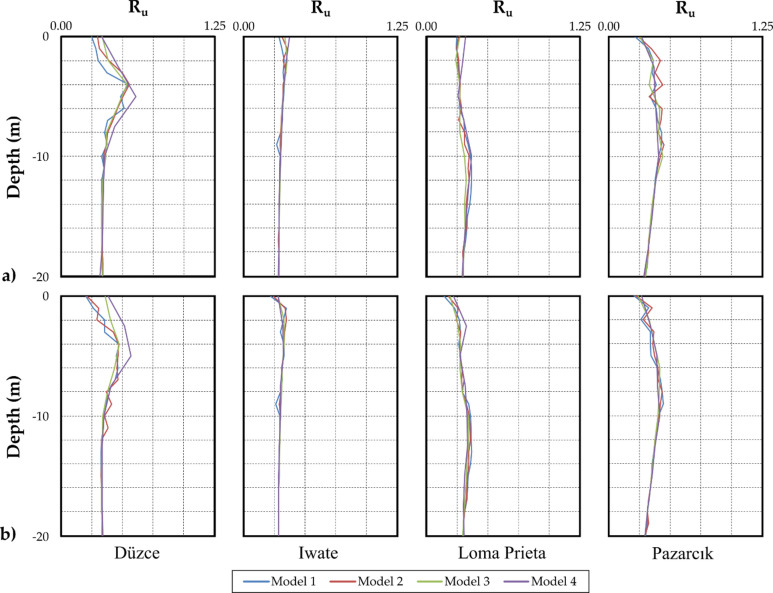
Variation of R_u_ with depth for a relative density of 75%: **a**) 6 nodes, **b**) 15 nodes.

By examining the R_u_ profiles from the ground surface to depth as presented in Figs. 8, 9 and 10, we highlight the critical roles of mesh refinement and element type across different soil densities. In Fig. 8, corresponding to the 35% relative density case, 6 nodes element analyses show that Models 1 and 2 yield nearly identical R_u_ profiles, while Model 4 exhibits pronounced deviations. Model 3 achieves intermediate accuracy, providing results closer to the fine-mesh references. When 15 nodes elements are employed, all mesh densities except Model 4 produce similar R_u_ values.

Figure 9 illustrates the 55% relative density scenario: with 6 nodes elements, Model 4 underpredicts R_u_ compared to Models 1–3, which remain closely aligned. Adopting 15 nodes elements enhances Model 4’s performance, leading to substantial overlap of R_u_ profiles across all mesh densities. In Fig. 10, for 75% relative density, both 6 and 15 nodes discretization yield virtually indistinguishable R_u_ values across all four mesh configurations. Overall, as relative density increases, even coarse meshes and 6 nodes elements provide sufficient accuracy; under loose sand conditions, however, finer meshes and fifteen-node elements are necessary to achieve the most consistent R_u_ predictions.

### Horizontal acceleration components at the ground surface

**Fig. 11:**
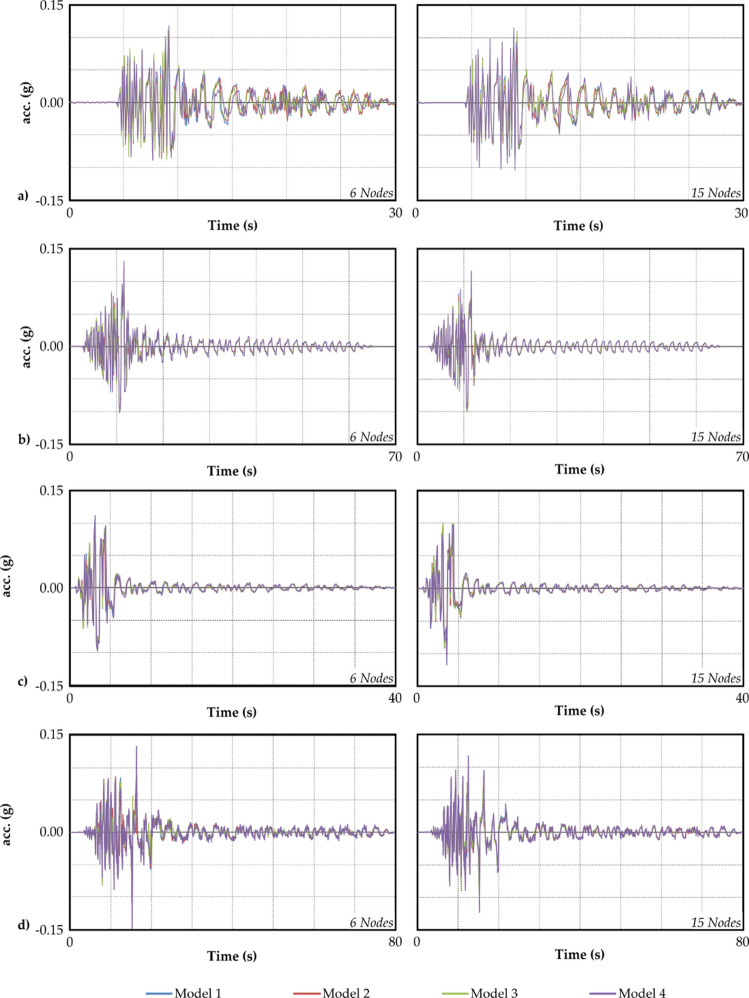
Surface horizontal acceleration components for a relative density of 35%: **a**) Düzce, **b**) Iwate, **c**) Loma Prieta, **d**) Pazarcık.

**Fig. 12:**
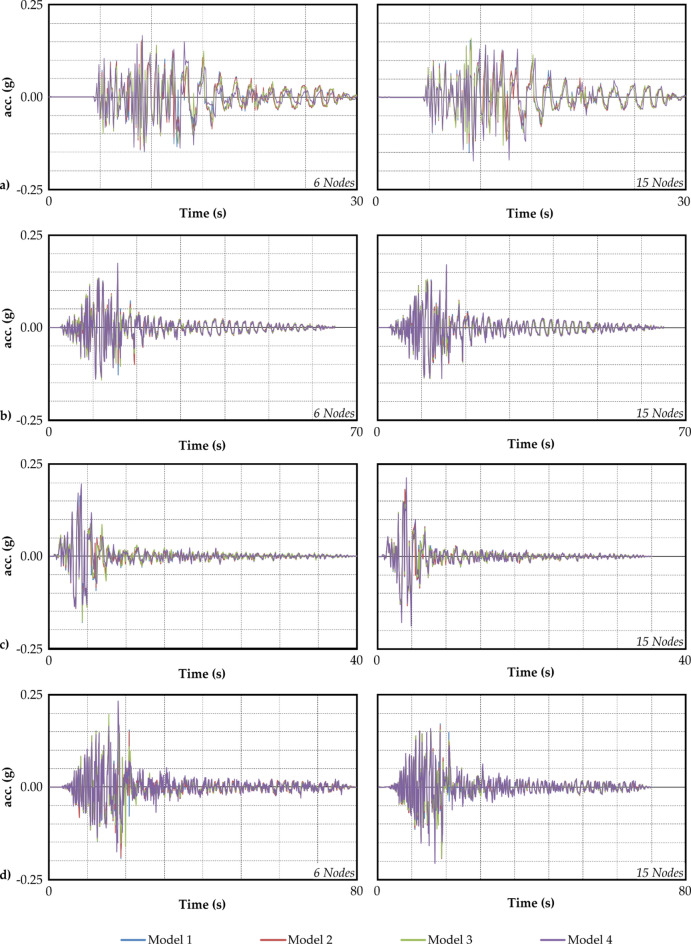
Surface horizontal acceleration components for a relative density of 55%: **a**) Düzce, **b**) Iwate, **c**) Loma Prieta, **d**) Pazarcık.

**Fig. 13:**
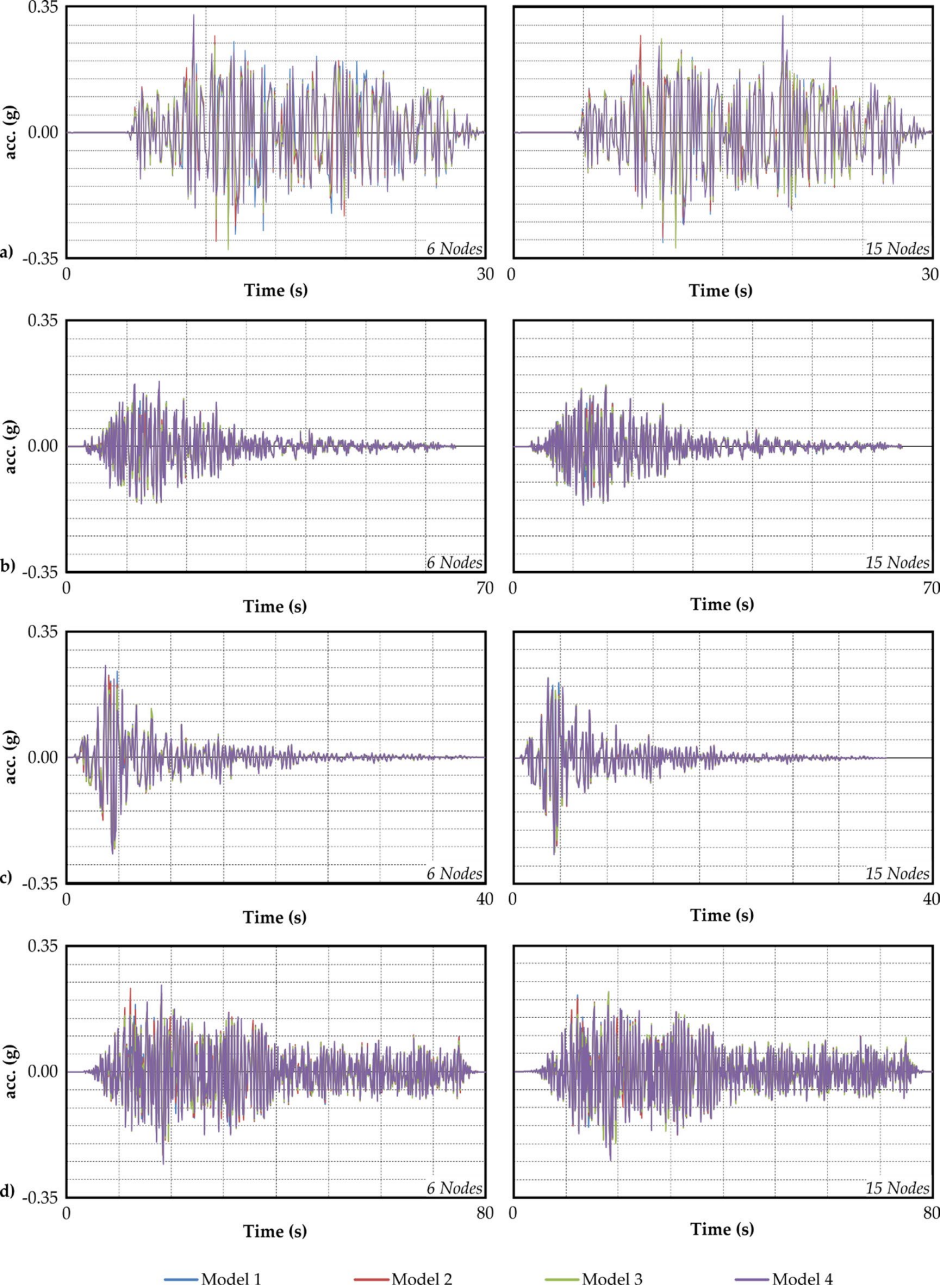
Surface horizontal acceleration components for a relative density of 75%: **a**) Düzce, **b**) Iwate, **c**) Loma Prieta, **d**) Pazarcık.

In order to assess the influence of finite-element mesh density and element type on the analysis outcomes, the horizontal component of the surface acceleration record was also examined. The results for different relative densities are shown in Figs. 11 and 12, and 13. In all cases, six-node results with the coarsest meshes (Models 3 and 4) show minor deviations from the reference traces, particularly in the Düzce and Loma Prieta records. Finer meshes (Models 1 and 2) produce nearly identical waveforms regardless of element type, indicating that element size is the primary factor controlling accuracy. Switching to fifteen-node elements reduces these deviations in the coarser meshes, yielding almost indistinguishable waveforms across all four mesh densities. This effect is most pronounced at 35% density (Fig. 11), where fifteen-node Model 3 aligns closely with Models 1 and 2. At higher densities (Figs. 12 and 13), all mesh and element combinations converge to the same waveform. These results demonstrate that while mesh refinement is essential for capturing detailed acceleration histories in loose conditions, the use of fifteen-node elements further improves consistency in coarse discretization. For medium-dense and dense sands, coarse meshes and six-node elements alone suffice to reproduce surface accelerations accurately, allowing computational savings without loss of reliability.

Table 5 demonstrates that maximum surface horizontal acceleration values vary systematically with mesh density, element type, and relative density. In all relative densities, coarser meshes (Model 4) produced the highest accelerations, whereas finer meshes (Models 1 and 2) yielded lower, tightly clustered peaks. Using 15 nodes elements reduces the gap between coarse and fine meshes, bringing all models into close agreement, although the variation for a given earthquake and relative density remains modest. At D_r_ = 75%, every mesh and element type combination converge to similarly acceleration values. These results indicate that in loose sands, the overestimation associated with coarse meshes can be mitigated by employing 15 nodes elements, while in dense sands even coarse meshes with 6 nodes elements achieve sufficiently accurate results.

**Table 5:** Maximum horizontal accelerations at the ground surface obtained from the different models.

D_*r*_ (%)	Element Type	Earthquake	Model 1	Model 2	Model 3	Model 4
35%	6 Nodes	Düzce	0.10	0.10	0.11	0.12
		Iwate	0.10	0.10	0.10	0.13
		Loma Prieta	0.10	0.10	0.10	0.11
		Pazarcık	0.12	0.13	0.13	0.15
	15 Nodes	Düzce	0.09	0.10	0.11	0.12
		Iwate	0.10	0.10	0.10	0.12
		Loma Prieta	0.10	0.10	0.10	0.12
		Pazarcık	0.11	0.11	0.11	0.12
55%	6 Nodes	Düzce	0.15	0.16	0.16	0.17
		Iwate	0.15	0.15	0.17	0.17
		Loma Prieta	0.18	0.18	0.18	0.20
		Pazarcık	0.19	0.19	0.20	0.23
	15 Nodes	Düzce	0.15	0.16	0.16	0.17
		Iwate	0.15	0.15	0.15	0.17
		Loma Prieta	0.18	0.18	0.18	0.21
		Pazarcık	0.19	0.19	0.19	0.21
75%	6 Nodes	Düzce	0.31	0.31	0.33	0.33
		Iwate	0.17	0.17	0.17	0.18
		Loma Prieta	0.26	0.26	0.26	0.27
		Pazarcık	0.23	0.23	0.25	0.26
	15 Nodes	Düzce	0.31	0.32	0.32	0.33
		Iwate	0.17	0.17	0.17	0.17
		Loma Prieta	0.26	0.26	0.26	0.27
		Pazarcık	0.23	0.23	0.23	0.25

### Pseudo spectral acceleration on surface

**Fig. 14:**
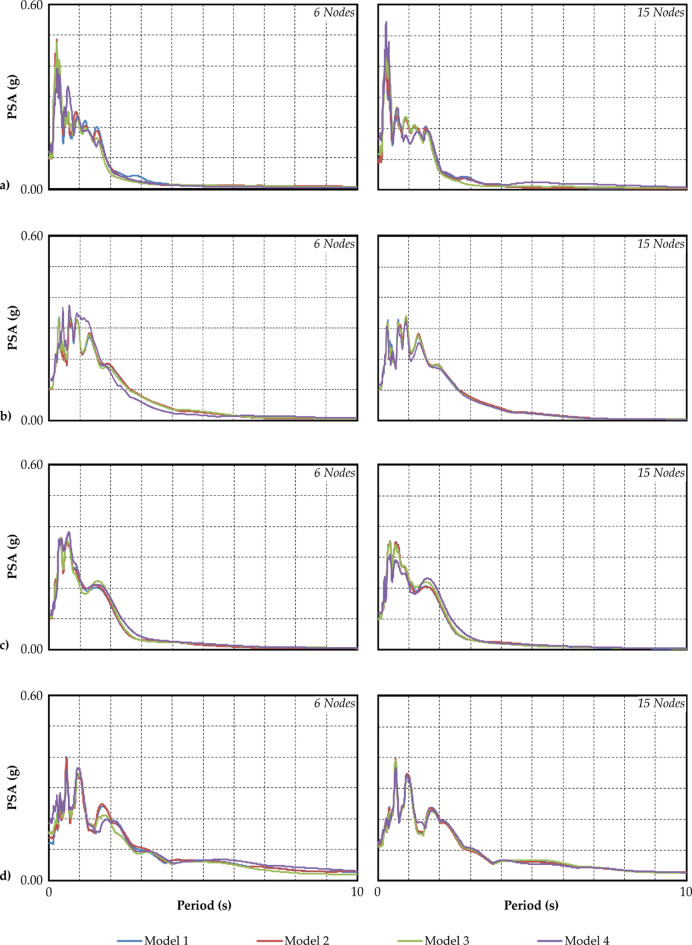
PSA values on surface for D_r_=35%: **a**) Düzce, **b**) Iwate, **c**) Loma Prieta, **d**) Pazarcık.

**Fig. 15:**
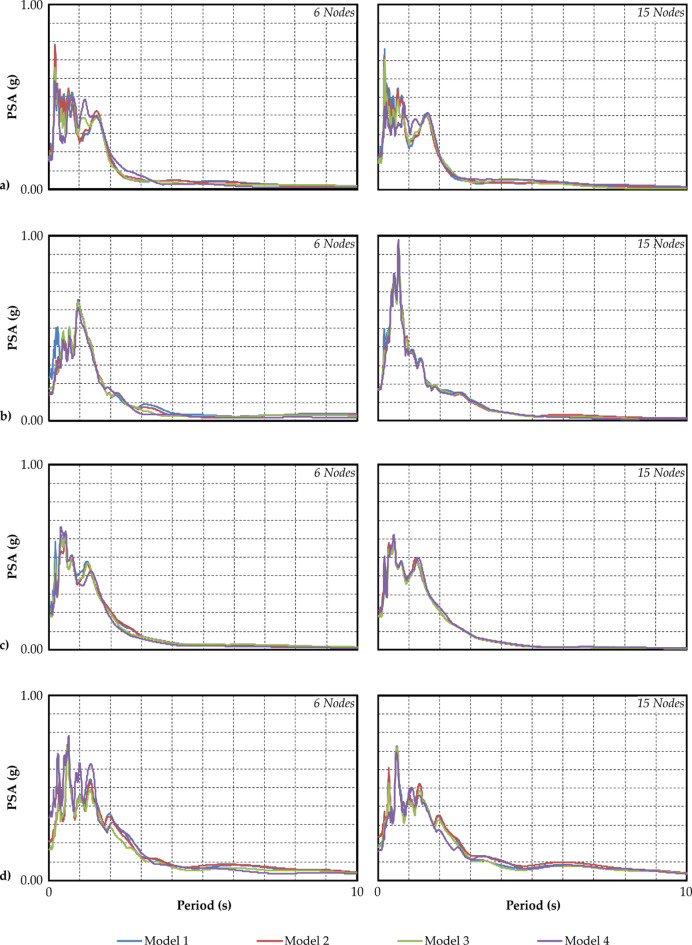
PSA values on surface for D_r_=55%: **a**) Düzce, **b**) Iwate, **c**) Loma Prieta, **d**) Pazarcık.

**Fig. 16:**
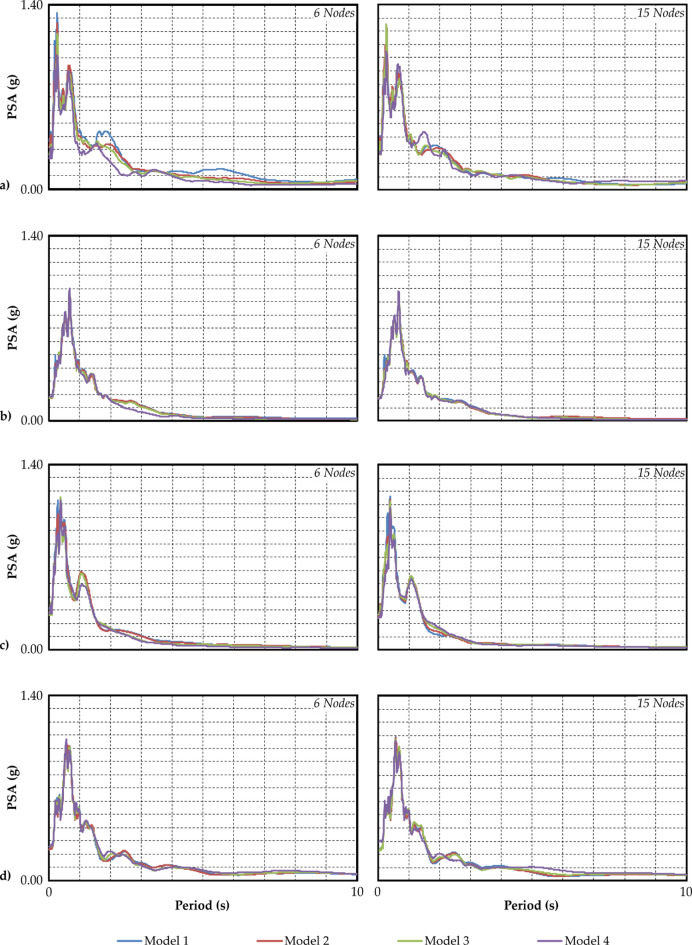
PSA values on surface for D_r_=75%: **a**) Düzce, **b**) Iwate, **c**) Loma Prieta, **d**) Pazarcık.

Figures 14, 15 and 16 present a comprehensive comparison of surface pseudo-spectral acceleration (PSA) responses for three representative soil stiffnesses (35, 55 and 75% relative densities). Each figure contrasts four progressively coarser mesh configurations (Models 1–4) and two element formulations (six-node versus fifteen-node) under identical seismic inputs. This arrangement allows direct assessment of how discretization parameters influence spectral demands at the ground surface, from highly deformable (loose) to nearly rigid (dense) sand conditions.

In the 35% relative density case (Fig. 14), six-node elements yield pseudo-spectral acceleration curves from Models 1 and 2 that nearly coincide, whereas Models 3 and 4 underpredict the peak values to some extent. When fifteen-node elements are employed, Model 3 converges almost exactly with the curves of Models 1 and 2, substantially reducing discrepancies; Model 4, however, still exhibits slight deviations. In the 55% relative density case (Fig. 15), the influence of mesh refinement further diminishes: six-node PSA traces cluster tightly across all four models, while fifteen-node elements virtually eliminate the minor deviations seen in coarser meshes. This behavior suggests that, for medium-dense sands, a coarser finite-element mesh may suffice to reproduce pseudo-spectral acceleration curves accurately. In the 75% relative density case (Fig. 16), both 6 and 15 nodes discretization yield virtually identical PSA curves across all four mesh densities. Consequently, whereas loose sands require fine finite-element meshes to accurately reproduce spectral demands, medium-dense and dense soils can be modeled with coarser meshes without compromising the precision of pseudo-spectral acceleration predictions.

### Computational efficiency and computation-time comparison

To quantitatively evaluate the trade-off between numerical accuracy and computational efficiency, the computation time of each dynamic analysis was recorded and compared for all mesh configurations. The analyses were performed on a workstation equipped with an AMD Ryzen 9 3900 × 12-Core Processor operating at 3.80 GHz, 32 GB RAM with a memory speed of 3200 MT/s, an AMD Radeon RX 6600 8 GB graphics card, and 3 TB of storage capacity. The reported computation times correspond to the dynamic calculation stage and are expressed in minutes. Pre-processing, model generation, meshing, and post-processing times were not included in the comparison.

Table 6 presents the computation times obtained for each relative density, element type, earthquake record, and mesh configuration. As expected, the computation time decreases consistently from Model 1 to Model 4 for all relative densities, earthquake records, and element formulations. This trend reflects the progressive reduction in the number of elements and nodes as the mesh becomes coarser. The results also show that 15-node elements require substantially longer computation times than 6-node elements for the same mesh density, particularly in the finer mesh configurations. This is attributed to the higher number of degrees of freedom associated with the higher-order interpolation scheme.

To further summarize the computational cost, the mean computation time was calculated for each relative density, element type, and mesh configuration by averaging the four earthquake records. The relative time was obtained by normalizing the mean computation time of each model by the corresponding Model 1 value for the same relative density and element type, where Model 1 was used as the reference case with a relative time of 1.00. The time saving was calculated as (1 - T_rel_) x 100, where T_rel_ is the relative computation time. For example, a relative time of 0.34 means that the model required 34% of the computation time of Model 1 and therefore achieved a 66% time saving. The summarized results are given in Table 7.

The summarized computation times confirm that mesh refinement is the dominant factor controlling the computational cost. For 6-node elements, the transition from Model 1 to Model 3 reduced the mean computation time by approximately 86%, 89%, and 93% for D_r_ = 35%, 55%, and 75%, respectively. For 15-node elements, the corresponding reduction from Model 1 to Model 3 was approximately 92%, 93%, and 92%. These reductions demonstrate the substantial computational benefit of using coarser meshes. However, the accuracy results presented in the previous sections show that the coarsest mesh configuration does not always provide reliable predictions, especially for loose sand and pore-pressure-related response quantities.

The comparison also highlights the practical role of 15-node elements. Although 15-node elements are computationally more expensive than 6-node elements for the same mesh density, their use with moderately coarse meshes can provide an efficient compromise. For example, the 15-node Model 3 configuration substantially reduced the computation time relative to the 15-node Model 1 configuration while maintaining better consistency with the fine-mesh benchmark than very coarse or lower-order discretizations. Therefore, the computation-time comparison supports the main accuracy-efficiency argument of the study: fine meshes remain preferable when pore-pressure-related quantities are critical, but higher-order elements combined with moderately coarse meshes may provide a practical alternative when both accuracy and computational efficiency are required.

**Table 6:** Computation times of the dynamic analyses for different mesh configurations.

D_*r*_ (%)	Element Type	Earthquake	Model 1 (min)	Model 2 (min)	Model 3 (min)	Model 4 (min)
35%	6 Nodes	Düzce	189	63	16	7
		Iwate	254	89	24	14
		Loma Prieta	135	47	12	3
		Pazarcık	469	159	95	17
	15 Nodes	Düzce	1032	237	82	15
		Iwate	1396	323	123	22
		Loma Prieta	737	174	62	12
		Pazarcık	2570	596	216	37
55%	6 Nodes	Düzce	237	65	20	7
		Iwate	211	78	25	4
		Loma Prieta	150	42	14	3
		Pazarcık	484	143	64	16
	15 Nodes	Düzce	1126	279	75	15
		Iwate	1153	309	89	18
		Loma Prieta	610	185	46	10
		Pazarcık	2114	573	165	32
75%	6 Nodes	Düzce	216	57	14	4
		Iwate	214	65	16	5
		Loma Prieta	119	36	9	4
		Pazarcık	347	103	26	7
	15 Nodes	Düzce	1004	238	78	13
		Iwate	995	238	84	16
		Loma Prieta	567	139	47	9
		Pazarcık	1592	394	136	24

**Table 7:** Mean computation time, relative time, and time saving for different mesh configurations.

D_*r*_ (%)	Element Type	Model	Mean Time (min)	Relative Time	Time Saving (%)
35%	6 Nodes	Model 1	262	1.00	-
		Model 2	90	0.34	66
		Model 3	37	0.14	86
		Model 4	10	0.04	96
	15 Nodes	Model 1	1434	1.00	-
		Model 2	333	0.23	77
		Model 3	121	0.08	92
		Model 4	22	0.01	99
55%	6 Nodes	Model 1	271	1.00	-
		Model 2	82	0.30	70
		Model 3	31	0.11	89
		Model 4	8	0.03	97
	15 Nodes	Model 1	1251	1.00	-
		Model 2	337	0.27	73
		Model 3	94	0.07	93
		Model 4	19	0.01	99
75%	6 Nodes	Model 1	224	1.00	-
		Model 2	65	0.29	71
		Model 3	16	0.07	93
		Model 4	5	0.02	98
	15 Nodes	Model 1	1040	1.00	-
		Model 2	252	0.24	76
		Model 3	86	0.08	92
		Model 4	16	0.01	99

### Practical recommendations and limitations

The comparisons of excess pore pressure histories, R_u_ profiles, surface acceleration records, and pseudo spectral acceleration responses show that mesh density and element order should not be selected independently of the soil state and the intended output of the analysis. The sensitivity of the numerical response to mesh refinement was more pronounced in loose saturated sand than in medium dense or dense sand, particularly for pore pressure related quantities. For the D_r_ = 35% cases, finer meshes or higher order elements are therefore recommended when the main objective is to evaluate excess pore pressure generation or the variation of R_u_ with depth. Under these conditions, very coarse discretization with 6 node elements should not be used for final design level assessments. A moderately coarse mesh combined with 15 node elements may, however, offer a reasonable compromise between numerical accuracy and computational efficiency.

For the D_r_ = 55% cases, the differences among the mesh configurations were less significant, especially in terms of surface acceleration and pseudo spectral acceleration. This suggests that moderately coarse meshes may be adequate for many practical applications, provided that the selected mesh still satisfies the relevant wave propagation requirements. The use of 15 node elements further improves the consistency of the response obtained from coarser meshes. For the D_r_ = 75% cases, the influence of mesh refinement became even less pronounced, and coarser meshes generally provided acceptable estimates of surface acceleration and spectral response. Nevertheless, caution is still required when local excess pore pressure accumulation, liquefaction triggering, or R_u_ profiles are the governing design quantities. For this reason, a mesh sensitivity check against a finer reference model is recommended before adopting a coarser discretization in design level analyses.

Although the prescribed Rayleigh damping was kept constant in all analyses, coarse spatial discretization may independently introduce numerical attenuation, numerical dispersion, and phase errors, particularly when the element size becomes large relative to the wavelength propagated through the soil profile. Therefore, in very coarse configurations such as Model 4, the computed response may reflect not only the common 2% Rayleigh damping but also discretization induced numerical effects. The results did not indicate a uniform over attenuation of the seismic response in the coarser configurations. Instead, the deviations were response dependent and were interpreted as the combined influence of numerical damping, wave dispersion, phase distortion, and the reduced resolution of nonlinear pore pressure generation. For this reason, the coarsest mesh configuration should be used with caution, particularly when pore pressure related quantities or liquefaction triggering are critical design outputs.

The recommendations presented in this section should be regarded as practical guidance rather than universal mesh size limits. They are derived from the numerical framework considered in this study, which consists of a 20 m saturated clean sand profile, three relative density states, four selected earthquake records, and the adopted PM4Sand calibration for Ottawa F 65 sand. The mesh size criteria used to define the numerical models are primarily governed by wave propagation requirements, including the shear wave velocity of the soil layer and the significant frequency content of the input motion, rather than by the sand type alone. Therefore, the qualitative influence of mesh density and element order observed in this study is expected to remain relevant for other saturated sand deposits, provided that the mesh size is selected consistently with the corresponding shear wave velocity profile and seismic input characteristics.

However, the quantitative response values and the magnitude of the differences among mesh configurations may depend on the adopted constitutive calibration. In particular, PM4Sand parameters such as G_0_ and h_p0_ may influence small strain stiffness, cyclic hardening behavior, pore pressure accumulation, and cyclic mobility. As a result, sands with different gradation, fabric, stiffness, dilatancy, contraction tendency, or cyclic resistance may exhibit different absolute values of excess pore pressure, R_u_, surface acceleration, and pseudo spectral acceleration. For deeper deposits, stratified profiles, strong impedance contrasts, sloping ground conditions, soil structure interaction problems, alternative boundary conditions, or different PM4Sand calibrations, the controlling wavelength and the relevant frequency range may differ from those examined here. In such cases, the mesh size should be selected with reference to the shear wave velocity profile, the significant frequency content of the input motion, and the response quantity of interest. The proposed recommendations should therefore be verified through project specific mesh sensitivity analyses when applied beyond the conditions investigated in this study.

## Conclusions

This study systematically investigated how finite element mesh density and element type influence the dynamic response of saturated sandy soils under seismic loading. Two dimensional finite element models were developed for three relative densities, namely D_r_ = 35%, 55%, and 75%, using four progressively coarser mesh configurations and both 6 node and 15 node triangular elements. Dynamic finite element analyses driven by four distinct earthquake records were performed to assess how variations in mesh density influence key numerical outcomes, including element quality, excess pore water pressure development, excess pore pressure ratio, surface horizontal acceleration, and pseudo spectral acceleration.

The results show that, under loose sand conditions, D_r_ = 35%, accurate numerical predictions are more sensitive to mesh refinement and element order. Coarse meshes with 6 node elements produced noticeable deviations in key response quantities, particularly in pore pressure related outputs, whereas the use of 15 node elements on moderately coarse meshes improved the agreement with fine mesh benchmark results. For medium dense sands, D_r_ = 55%, the differences among the mesh configurations became less pronounced, and the use of 15 node elements further reduced the remaining discrepancies in coarser discretizations. For dense sands, D_r_ = 75%, the responses obtained from different mesh densities and element types converged to nearly identical predictions under the conditions examined in this study.

From a practical perspective, the findings suggest that mesh selection should be made according to both the relative density of the sand and the response quantity of interest. For loose saturated sands, finer meshes or higher order elements are recommended, particularly when excess pore pressure generation and R_u_ profiles are the main outputs. For medium dense and dense sands, coarser meshes may provide sufficiently accurate estimates of surface acceleration and pseudo spectral acceleration, provided that the mesh satisfies the relevant wave propagation requirements. Nevertheless, very coarse meshes should be used with caution when pore pressure related quantities or liquefaction triggering are critical for design.

The applicability of the proposed recommendations should be interpreted in relation to the wave propagation criteria used to define the average element size. Although the analyses were performed for a 20 m saturated clean sand profile, the average element size is not governed by the profile depth alone, but primarily by the shear wave velocity profile, the controlling wavelength, and the significant frequency content of the input motion. Therefore, the qualitative findings regarding the influence of mesh density and element order are expected to remain relevant for other saturated sand profiles when the mesh size is selected consistently with the corresponding wave propagation requirements. However, deeper deposits, stratified soil profiles, strong impedance contrasts, sloping ground conditions, soil structure interaction problems, or more complex boundary conditions may alter the controlling wavelength, modal characteristics, and relevant frequency range of the system. For such cases, the recommendations presented in this study should be regarded as practical guidance rather than universal mesh size limits, and at least one project specific mesh sensitivity analysis against a finer reference model is recommended for soil profiles, constitutive model calibrations, and boundary conditions different from those considered herein.

## Data Availability

Data Availability StatementAll data generated or analysed during this study, including numerical model parameters, boundary conditions, mesh configurations, and analysis results, are included in this published article.
